# Osseointegration of standard and mini dental implants: a histomorphometric comparison

**DOI:** 10.1186/s40729-017-0079-1

**Published:** 2017-05-01

**Authors:** Jagjit S. Dhaliwal, Rubens F. Albuquerque, Monzur Murshed, Jocelyne S. Feine

**Affiliations:** 1 Faculty of Dentistry, McGill University, 2001 McGill College Avenue, Suite 500, Montreal, Quebec H3A 1G1 Canada; 2 Faculty of Dentistry of Ribeirão Preto, University of São Paulo, Ribeirão Preto, SP Brazil; 3 Department of Medicine, McGill University, Montreal, Quebec Canada

**Keywords:** Bone implant contact, Mini dental implant, Osseointegration

## Abstract

**Background:**

Mini dental implants (MDIs) are becoming increasingly popular for rehabilitation of edentulous patients because of their several advantages. However, there is a lack of evidence on the osseointegration potential of the MDIs. The objective of the study was to histomorphometrically evaluate and compare bone apposition on the surface of MDIs and standard implants in a rabbit model.

**Methods:**

Nine New Zealand white rabbits were used for the study to meet statistical criteria for adequate power. Total 18 3M^™^ESPE^™^ MDIs and 18 standard implants (Ankylos^®^ Friadent, Dentsply) were inserted randomly into the tibia of rabbits (four implants per rabbit); animals were sacrificed after a 6-week healing period. The specimens were retrieved en bloc and preserved in 10% formaldehyde solution. Specimens were prepared for embedding in a light cure acrylic resin (Technovit 9100). The most central sagittal histological sections (30–40 μm thick) were obtained using a Leica SP 1600 saw microtome. After staining, the Leica DM2000 microscope was used, the images were captured using Olympus DP72 camera and associated software. Bone implant contact (BIC) was measured using Infinity Analyze software.

**Results:**

All implants were osseointegrated. Histologic measures show mineralized bone matrix in intimate contact with the implant surface in both groups. The median BIC was 58.5 % (IQR 8.0) in the MDI group and 57.0 % (IQR 5.5) in the control group (*P >* 0.05; Mann-Whitney test). There were no statistical differences in osseointegration at 6 weeks between MDIs and standard implants in rabbit tibias.

**Conclusions:**

Based on these results, it is concluded that osseointegration of MDIs is similar to that of standard implants.

## Background

The term “osseointegration” was first introduced to explain the phenomenon for stable fixation of titanium to bone by Brånemark et al. in the 1960s [1]. Osseointegrated implants were introduced, a new era in oral rehabilitation began, and many studies were conducted [2, 3]. A success rate of over 90% has been reported [4, 5]. Further, a success rate of 81% in the maxillary bone and 91% in the mandible can be accomplished [6]. Dental implants have been widely used for the stabilization of complete dentures and also help to maintain bone, function, esthetics, and phonetics and improve the oral health-related quality of life [7]. The dental implants are available with different surfaces and sizes. The size of the dental implants usually ranges in the diameter range of 3 mm (narrow diameter) to 7 mm (wide diameter). However, majority of the implants fall in the “standard diameter” range of 3.7 to 4.0 mm [8].

Mini dental implants or small size implants are also being widely used for stabilizing the complete dentures [9], for orthodontic anchorage [10–12], single tooth replacements [13, 14], fixing the surgical guides for definitive implant placement [15], and as transitional implants for the support of interim removable prosthesis during the healing phase of final fixtures [16, 17].

The single-piece mini dental implants (MDIs) are becoming increasingly popular for the purpose of denture stabilization. There are many advantages of the MDIs over the regular implants. The surgery is minimally invasive as compared with conventional implant surgery which helps in decreased morbidity for the patient. Transmucosal placement is possible using a single pilot drill, and these can often be loaded immediately [18]. Gingival healing is typically seen in 2 to 5 days, extended healing period with MDIs is usually not necessary [19]. The insertion of MDIs needs a minimal disturbance of the periosteum, thus osseointegration process is accelerated and time needed for MDIs tends to be considerably small than that of regular implants due to less injurious insertion procedure [9]. The need for sutures or long recovery periods is eliminated [3]. The patient can walk in to the office in the morning and is out the same day with a full set of teeth, the patient is allowed to eat the same day. These can work well for patients who have significant bone loss that restrict them from being a candidate for regular dental implants. MDIs are also a solution for patients that cannot have surgery for medical reasons. MDIs are also cost effective [20]. Considerable confusion exists in the literature regarding the best method to monitor the status of a dental implant. Various methods have been used to demonstrate the osseointegration of dental implants. A common and time-tested method to evaluate biological responses to an implant is to measure the extent of bone implant contact (BIC), referred to as histomorphometry at the light microscopic level. Bone implant contact (BIC) is one of the parameters which has been used extensively to study the amount of bone apposition next to the implants [21–27]. When an implant is placed in the jaw, it is in contact with compact bone as well as cancellous bone. The different structures of the two types of bone frequently result in variation of mineralized bone-to-implant contact length along the implant surface [28, 29]. Albrektsson et al. identified the key features affecting osseointegration about 4 decades ago, e.g., implant surface and topography, surface chemistry, charge, and wettability [30]. Roughness and enhanced surface area seems to be helpful for osseointegration. Carlsson et al. reported that screw-shaped implants with a rough surface had a stronger bonding than implants with a polished surface [31]. A coarse surface seems to be more appropriate for osseointegration of implants than a relatively smoother implant surface by representing a greater degree of implant integration [32–34]. The bone contact areas of 3M^™^ESPE^™^ MDIs are surface treated. The treatment process of these MDIs includes sandblasting with aluminum oxide particles followed by cleaning and passivation with an oxidizing acid [35].

Despite the advantages of the mini dental implants, evidence on their efficacy and long-term success is lacking. The success of these implants will depend on their union with the surrounding bone. New implant systems entering into the market have to be studied with the help of animal models first, to demonstrate the osseointegration potential for their probable success in humans. There is a limited evidence regarding the 3M^™^ESPE^™^ MDIs. Therefore, there is a need for an animal study to explore the osseointegration of these implants to assist in better understanding of the treatment selection, prognosis, and outcomes for the patients.

### Objectives of the study

The objective of this study is to compare bone apposition on the surface of mini dental implants and standard implants by means of histomorphometric methods.

## Methods

### Animal model

Nine clinically healthy New Zealand white rabbits weighing 3.5 kg and more were used for the study, and the animals were housed in the central animal house facility. The head of tibia/femur of the animals were used for the implantation of samples. Rabbits’ tibiae and femur have been widely used as an animal model by various other authors to study osseointegration of dental implants [36–45].

### Sample size

The sample size of this study has been calculated based on the results of a similar study by Bornstein et al. [22]. It was established that 88% statistical power will be achieved by using 18 mini dental implants (3M^™^ESPE^™^ MDIs) for the experimental implants and equal number of an established regular implant (Ankylos^®^, Dentsply Friadent GmbH) for the control. Therefore, the total number of implants used was 36. Each animal received four implants on hind limbs, i.e., right and left tibia/femur head randomly (the heads of tibia and femur have been chosen to get the maximum bulk of bone). Therefore, each animal received two experimental and two regular implants.

### Surgical procedure

The procedures were approved by the institutional animals’ ethics review board of McGill University, Canada. Animals were anesthetized by an intravenous injection of ketamine hydrochloride-xylazine mixture at 35–50 and 1–3 mg/kg respectively according to a method described by Green et al. [46]. Acepromazine was injected subcutaneously at dosage of 1 mg/kg. Further injections of the mixture were given to maintain anesthesia, if necessary [46]. Sterile ophthalmic ointment was put in both eyes to prevent corneal desiccation. Animals were shaved for twice the size of the expected surgical field with an electric razor. All loose hair and debris from the animal were removed. The surgical area was cleaned with gauze and 2% chlorhexidine solution to remove the majority of debris from the surgical site. Antiseptic skin preparation was done starting at the center of the surgical site and moved to the outside of the prepared area in a circular manner. Three scrubs with 2% chlorhexidine solution and three alternating rinses with alcohol were performed. The animal was draped and fixed with clamps on a sterile, impermeable covering to isolate the disinfected area. This was performed by the gloved and gowned surgical team under sterile conditions.

### Surgical protocol for 3M^™^ESPE^™^ MDIs

A small longitudinal skin incision just distal to the tibia-femur joint was made. The tibia/femur head was exposed subperiosteally and an osteotomy performed with the delicately placed pilot drill over the entry point and lightly pumped up and down under copius saline irrigation just to enter the cortical bone for the MDIs. This was used for initial bone drilling to depth of 0.5 mm. The 3M^™^ESPE^™^ MDI (size 1.8 mm × 10 mm) vial was opened and the body of the implant was firmly grasped with a sterilized locking pliers. The titanium finger driver was attached to the head of the implant. The implant was transferred to the site and rotated clockwise while exerting downwards pressure. This began the self tapping process and was used until noticeable bony resistance encountered when it touched the lower cortical plate. The winged thumb wrench was used for driving the implant deeper into the bone, if necessary. All the animals received one MDI on the head of each tibia or femur. Therefore, total 18 mini dental implants were inserted.

### Surgical protocol for the Ankylos^®^ implants

Equal number of comparator implants (size 3.5 mm × 8 mm) were inserted in the other tibia/femur head of the animals after doing the osteotomy according to the manufacturer’s protocol as follows. After mobilizing the mucoperiosteal flap, the 3-mm center punch was used to register a guide for the twist drill. The twist drill was used to establish the axial alignment of the implant and to assist in the guidance of the depth drill. The depth drills were sequentially used to create osteotomy to the subcrestal axial depth of 0.5 mm. The conical reamer was used to develop the conical shape of the implant body and to check the osteotomy depth. A counter-clockwise rotation was used to compress the bone in soft bone. The tap or thread cutter was used for dense bone to create the threads in the osteotomy. The thread cutter’s diameter corresponds to the implant diameter. To engage the implant into the implant placement tool, the square faces on the implant fixture mount were aligned with those on the implant placement tool, then pushed together. Using the handle (finger wheel), the implant was pulled out of inner vial and the plastic collar was discarded. The implant placement assembly was transferred to the osteotomy and the implant was secured into the osteotomy site. The implant placement was started with the handle and finally placed using the hand-ratchet. If excessive force was experienced, the osteotomy was rinsed out and the depth was checked by retapping. To disengage fixture mount from implant, the open-ended spanner was used to break the retention force of the fixture mount retention screw. The knurled top of the implant placement tool was turned by hand to fully disengage the fixture mount with the implant. Pushing down on the knurled top of the implant placement tool disengaged the fixture mount.

### Suturing

Expected length of the procedure was approximately 1 h. Following placement of the implants, the wound was sutured in layers. The underlying muscle, fascia, and dermal layers were sutured with the help of Vicryl (Polyglactin 910) suture with 3/8 circle reverse cutting needle. The skin was sutured to a primary closer with the same suture material.

### Radiograph

**Fig. 1:**
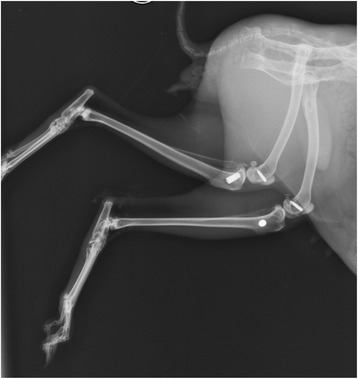
Radiograph showing implants in the rabbit tibia

Plain X-ray images of all the rabbit tibia were taken after suturing to confirm the position of implants and to detect any injury/fracture of the bone (Fig. 1).

### Post surgical treatment

After the surgical procedure, the animals were housed in a cage under the supervision of a veterinary doctor until they came out of anesthesia. The rabbit was observed every 2 h on the first day of surgery followed by once a day to check the wound for infection. The wound was protected with povidone iodine ointment. The rabbits were allowed immediate weight bearing as tolerated; therefore, they had no restraints on weight bearing.

Animals were shifted and housed together with other rabbits. The rabbit was given a dose of Cephalexin 12 mg/kg 0.5 ml I.V. once intraoperatively and a postoperative analgesic, i.e., Carprofen 2–4 mg/kg S.C. every 8 hourly for 3 days according to McGill SOP. The routine daily care was as per McGill SOP#524.01.

The feeding protocols were followed according to the university central animal house facility guidelines. The animals had a free access to water and feed. The sutures were removed after 7–10 days, and the wound was cleaned with 0.2% chlorhexidine solution.

### Euthanasia

The animals were euthanized at 6 weeks respectively. An overdose of pentobarbital sodium 1 ml/kg intravenously, under general anesthesia, was used for this purpose [47, 48].

### Specimen retrieval

The implants along with their surrounding bone were excised with a surgical saw right away following the euthanasia. The excess tissue was dissected and the specimens were removed en bloc with a margin of surrounding bone of about 5–10 mm. The specimens were immediately put into the 10% formaldehyde solution.

### Sample preparation for embedding

The specimens were dehydrated in the ascending graded ethanol solution and kept in a pre-filtration solution for 3 h at room temperature and then in the filtration solution at 4 °C for 17 h. The specimens were then embedded in a light curing resin Technovit 9100 NEW (Kulzer & Co., Wehrheim, Germany) polymerization system based on methyl methacrylate, specially developed for embedding mineralized tissues for light microscopy. The polymerization mixture was produced by mixing the solution A and B in the proportion of 9 parts A and 1 part of solution B directly before use. This was done in a beaker and using a glass rod to stir the mixture. The samples were then positioned in the labeled plastic moulds, completely covered in the polymerization mixture, and placed in cooled desiccators and under a partial vacuum at 4 °C for 10 min. The resulting blocks were placed in a sealed container and left to polymerize between −8 and −20 °C. The samples were allowed to stand at 4–8 °C in the refrigerator for at least 1 h before allowing it to slowly come to room temperature. The polymerization times are dependent on the volumes of polymerization mixture used and of the constancy of the temperature at which polymerization is carried out.

### Preparation of histological sections

**Fig. 2:**
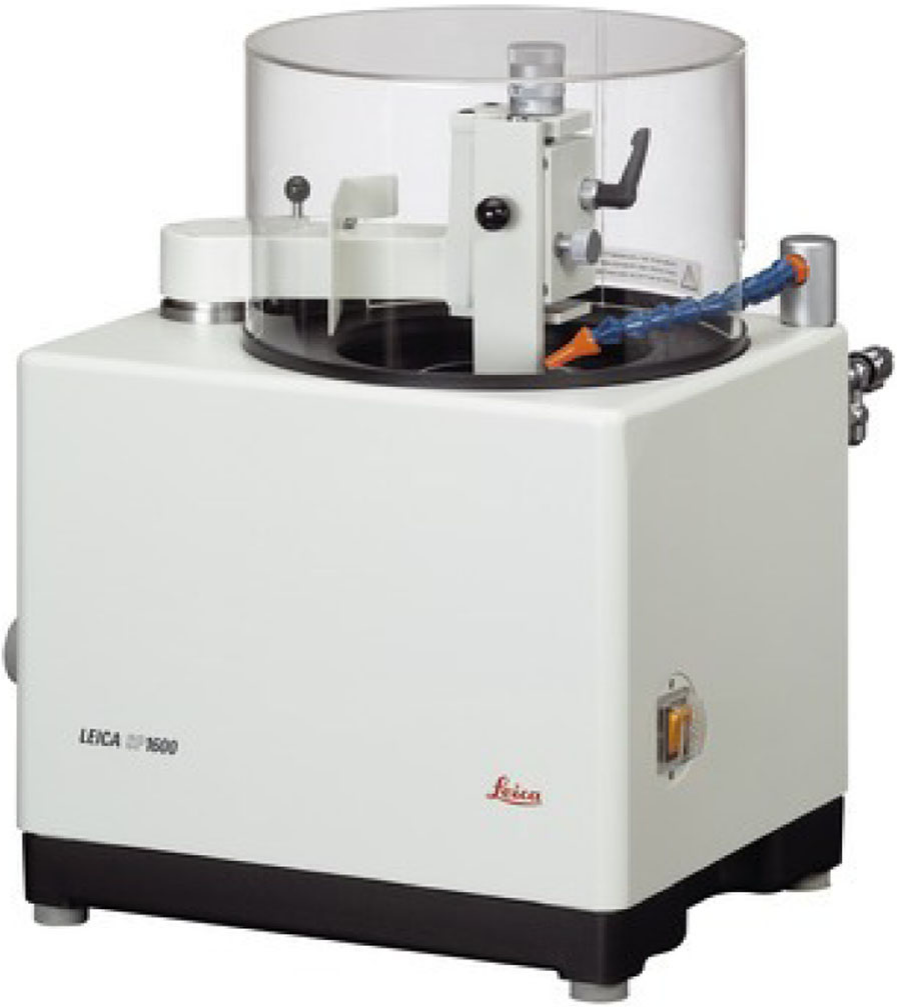
Leica SP 1600 saw microtome

**Fig. 3:**
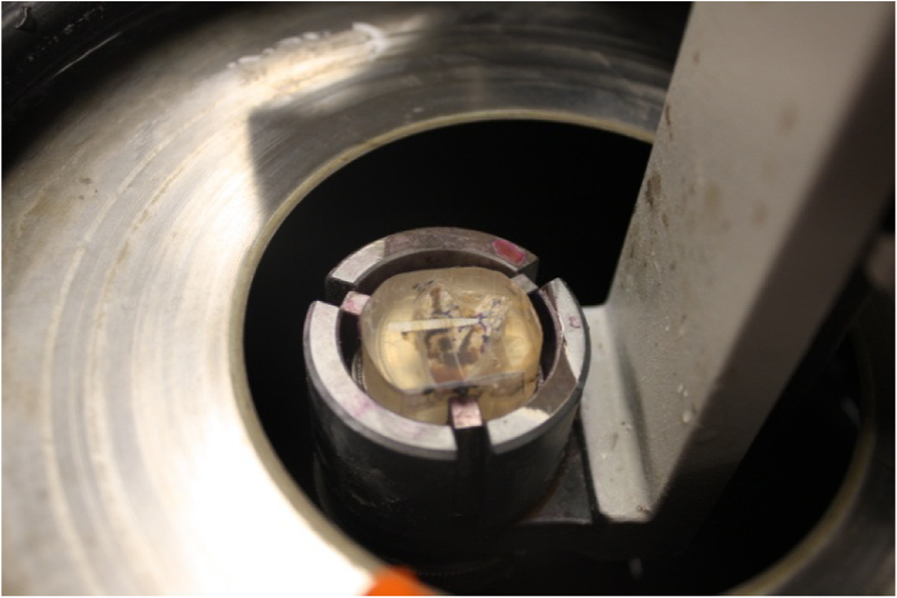
Histological sections being obtained with Leica SP 1600 saw microtome

The acrylic block was mounted into the object holder of the Leica SP 1600 saw microtome (Fig. 2). The height of the object was adjusted until the surface of the object is slightly above the upper edge of the saw blade. The surface of the block was trimmed to get a plane surface prior to producing slices of a defined thickness. During the sawing process, the water flow was adjusted so that the water jet lands on the edge of the saw blade. The built-in water cooling device prevents overheating of the object and removes saw dust from the cutting edge and thus prolongs the lift time of the saw blade. The most favorable feed rate was determined (Fig. 3). After trimming, the first undefined slice was removed from the saw blade. The desired section thickness was selected, considering the thickness of the saw blade and added to the desired thickness of final section. The section was stabilized during the sawing process. To do so, a glass cover slip was glued onto the trimmed surface of the specimen block using cyanoacrylate glue. These blocks were cut with a low speed saw under water along the lateral surface of the implant [47, 48]. The implant bearing blocks were cut parallel to the long axis of the implant, and 30-μm-thick specimens were obtained.

The saw blade has a thickness of 280 μm and a feed of 310 μm was selected to obtain the final section thickness of 30 μm. The knurled screw was used for the setting of the section thickness. The prepared section was finally removed from the saw blade. The specimens were prepared for histology by the method as described by Donath and Breuner [49].

### Histological evaluation

Subsequently, the sections were stained with toluidine blue and basic fuchsin similar to other studies [21, 22, 50]. The specimen sections were evaluated at the most central saggital section of each implant under an optical microscope after staining. The images were photographed with a high resolution camera and interfaced to a monitor and PC, observed under the Leica DM2000 microscope, and the images were captured using Olympus DP72 camera and associated software [4, 21, 22]. Bone implant contact (BIC) was measured using Infinity Analyze software. Six images of the same implant were taken and measurements were done. The percentage of the interface contact length between implant surface and bone, i.e., bone implant contact (BIC), was calculated. The percentage of bone tissue in a 200-μm-wide zone parallel to the contour of the implant area (adjoining the implant) was measured.

### Micro-computed tomography (MicroCT)

MicroCT scans of each sample of both types of implants were obtained with a Skyscan 1172 equipment (Kontich, Belgium) at 6 μm resolution with 800 ms exposure time, 70 kV electric voltage, 167 μA current, and a 0.5-mm thickness aluminum filter. The equipment was fitted with a 1.3-MP camera to capture high resolution 2D images that were assembled into 3D reconstructions using NRecon software supplied with the instrument.

### Statistical methods

Mean values and standard deviations were calculated for bone implant contact (BIC). Univariate analysis was done for all the evaluations. Analysis of variance (ANOVA) was used to analyze the differences between the two implants. *P* value <0.05 was considered significant. Statistical analyses were carried out with the help of SPSS statistical software version 18.

## Results

### Clinical findings

On the whole, postoperative wound healing in all the rabbits was good. None of them exhibited any signs of wound infection or exposure. A total of 36 specimens were retrieved for histological examination.

### Histological observations

**Fig. 4:**
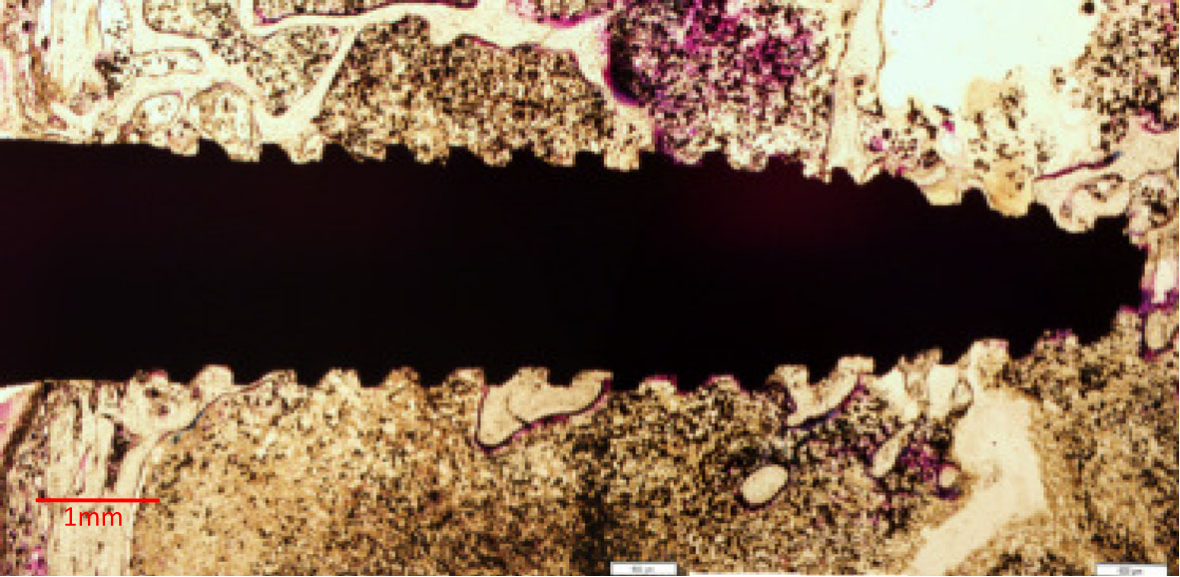
Histological section of mini dental implant in rabbit tibia stained with methylene blue and basic fuchsin

**Fig. 5:**
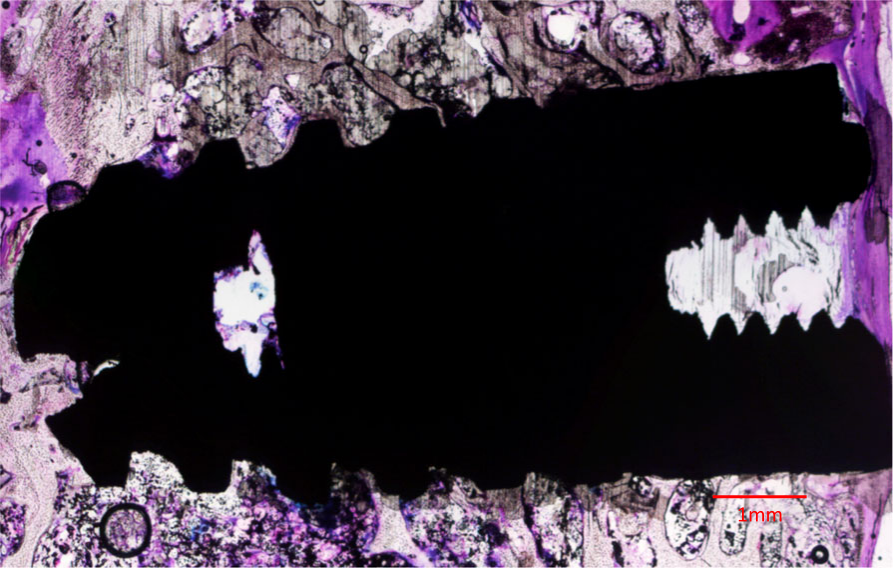
Histological section of standard implant in rabbit tibia stained with methylene blue and basic fuchsin

**Fig. 6:**
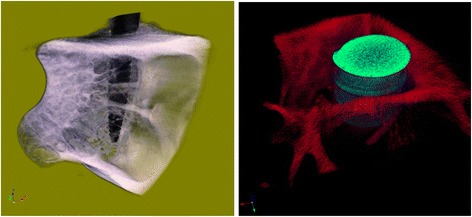
Micro CT scan images of the MDIs and Ankylos^®^ embedded in rabbit bone 6 weeks post implantation

All of the implants in both groups showed osseointegration and displayed a good amount of bone contact length (Figs. 4 and 5). No discernible differences were noticed between both the groups. The zone of interest was 200 μm in the peri-implant area of the implants on both sides. Due to large marrow spaces in the rabbit bone, larger volume of bone contact was mostly observed in the coronal and apical portions of the implants. The MicroCT pictures showed a three-dimensional deposition of bone in both samples (Fig. 6). It was noted that possibility of new bone formation was higher in areas adjacent to old bone. The sections of implant, which were exposed to the marrow spaces, displayed either no bone deposition or very thin bone tissue. Newly formed bone was seen with lighter staining. In the surrounding areas of both types of implants, bone fragments were noticed around the implant. These could correspond to bone fragments during the osteotomy procedure. Percentage of BIC ranged from 45 to 67% in both the groups. The median value of % BIC was 58.5 and the MDI group (IQR 7) and control group was 57.0 (IQR 5.0) (Tables 1 and 2). The mean differences of % BIC between the groups were verified through Mann–Whitney nonparametric test. There was no significant difference between the % bone implant contact (BIC) length of both the implants (*P* value >0.05).

**Table 1:** Comparison of % BIC in both groups

Sample	3M^™^ESPE^™^ MDIs	Ankylos^®^
1.	67	54
2.	59	67
3.	54	45
4.	51	58
5.	47	57
6.	64	49
7.	50	54
8.	60	56
9.	56	60
10.	61	53
11.	62	59
12.	61	55
13.	59	59
14.	45	51
15.	58	59
16.	54	62
17.	66	62
18.	56	57

**Table 2:** Descriptive statistics of the experimental and control group

BIC	3M^™^ ESPE^™^ MDIs	Ankylos^®^ Friadent (Dentsply)
Median	58.5	57
Mean	57	56.5
Interquartile range	8	5.5
First quartile	53.25	53.75
Third quartile	61.25	59.25

## Discussion

The osseointegration potential of 3M^™^ESPE^™^ MDIs has not been studied. The MDI is a one-piece implant that simplifies the restorative phase resulting in a reduced cost for the patient. Titanium-aluminum-vanadium alloy (Ti 6Al-4V-ELI) is used for increased strength. The success of these implants led to its use in long-term fixed and removable dental prostheses [51]. Conventional implant treatment requires adequate bone width and interdental space. Augmentation procedures are complex and can cause postoperative pain and discomfort for the patient and additional costs.

In human models, a 3–6-month period is needed to obtain osseointegration and animal models would need a shorter time (4–6 weeks) [30, 33]. Rabbit has been used extensively to examine osseointegration and appears to be an appropriate model for studying the bone healing systems [52]. The healing periods used by various authors for assessing the bone implant contact in rabbits are 2, 3, 4, 6, 8, and 12 weeks [53–57]. However, the best results have been between 6 and 12 weeks of insertion period [51, 53–55]. The 6-week healing period was carefully chosen after literature search. This was in agreement with others who have reported that a 6-week period is adequate in rabbits to develop a “rigid osseous interface” [51–60].

At the bone implant interface, woven bone starts forming after the placement of implant. Lamellar bone slowly replaces this scantily organized bone. The fully developed lamellar bone which replaces the woven bone typifies a stable and lasting osseointegration [61].

Our results are in concurrence with Balkin et al. [62]; they have also shown in their histology study in humans that the MDI undergoes osseointegration. They inserted one 3M^™^ESPE^™^ MDI of 1.8-mm diameter in each of two patients as a transitional implant for mandibular dentures. After a period of 4 and 5 months, the implants were trephined out for histological evaluation. The results showed that there was a close apposition of bone on the implant surfaces. The bone surrounding the implant demonstrated signs of matured healing and integrated for immediate function after 4 to 5 months of healing period.

Our study is also in concordance with the results of a removal torque study by Simon et al. [63] in immediately loaded “transitional endosseous implants” in humans. The percentage BIC for MDIs was similar to standard implants.

The surface topography also affects the BIC, Wennerberg et al. [32] measured and compared removal torque values on screw-shaped titanium implants with three surface types. The results showed that screws sandblasted with 25-μm particles of titanium and 75-μm particles of aluminum oxide exhibited a higher removal torque and interfacial bone contact than the machined titanium implants with smoother surface texture.

The surface of 3M^™^ESPE^™^ MDI is sandblasted with aluminum oxide and cleaned and passivized with an oxidizing acid (Technical Data Sheet, 3M ESPE) [35]. The surface of Ankylos^®^ is sandblasted and acid etched [64]. Various authors have reported that surface roughness induces a variety of events in the course of osteoblast differentiation, spreading and proliferation, production of alkaline phosphatase, collagen, proteoglycans, and osteocalcin, and synthesis of cytokines and growth factors [65–67]. Therefore, leading to bone deposition on the surface of these implants, Yan et al. [68] demonstrated that simple surface treatments can turn the titanium surface into a bone-bonding one. With the results of our in vitro study, Marulanda et al. [69] on discs of both types of implants demonstrated that surface chemistry of 3M^™^ESPE^™^ MDI is conducive to growth of osteoblasts leading to bone apposition.

One of the shortcomings of our study may be the use of rabbit tibia as a model. The tibia of the rabbit is essentially hollow except the upper and lower cortical plates. This may justify lack of bone apposition on the whole implant in both experimental as well as comparator implants. However, it provides a reliable information for human application as the human maxillary bone is also of a softer bone quality [36, 51].

## Conclusions

The results of this study show that MDIs as well as regular implants osseointegrate in rabbits.
