# Erratum to: Osseointegration of standard and mini dental implants: a histomorphometric comparison

**DOI:** 10.1186/s40729-017-0088-0

**Published:** 2017-06-28

**Authors:** Jagjit S. Dhaliwal, Rubens F. Albuquerque, Monzur Murshed, Jocelyne S. Feine

**Affiliations:** 1 Faculty of Dentistry, McGill University, 2001 McGill College Avenue, Suite 500, Montreal, Quebec H3A 1G1 Canada; 2 Faculty of Dentistry of Ribeirão Preto, University of São Paulo, Ribeirão Preto, SP Brazil; 3 Department of Medicine, McGill University, Montreal, Quebec Canada

## Erratum

Upon Publication of the original manuscript [1] several discrepancies were highlighted in the following sections; Statistical methods and Results. These errors have since been acknowledged and corrected in this erratum.

**The text in the subsection “Statistical methods” originally read:**

“Mean values and standard deviations were calculated for bone implant contact (BIC). Univariate analysis was done for all the evaluations. Analysis of variance (ANOVA) was used to analyze the differences between the two implants. P value <0.05 was considered significant. Statistical analyses were carried out with the help of SPSS statistical software version 18.”

**This should read:**

“Mean values and standard deviations were calculated for bone implant contact (BIC). The mean differences of % BIC between the groups were verified through a Mann–Whitney nonparametric test, *P* value <0.05 was considered significant. Statistical analyses were carried out with the help of SPSS statistical software version 18.”

**The final paragraph of the results section reads:**

The median value of% BIC was 58.5 and the MDI group (IQR 7) and control group was 57.0 (IQR 5.0) (Tables 1 and 2).The mean differences of % BIC between the groups were verified through Mann–Whitney nonparametric test. There was no significant difference between the % bone implant contact (BIC) length of both the implants (*P* value >0.05).

**This should read:**

Percentage of BIC ranged from 45 to 67% in both the groups. The median value of % BIC was 58.5, MDI group (IQR 8), and control group was 57.0 (IQR 5.5) (Tables 1 and 2). The mean differences of % BIC between the groups were verified through a Mann–Whitney nonparametric test. There was no significant difference between the % bone implant contact (BIC) length of both the implants (*P* value >0 .05).
